# Analyte-mediated formation and growth of nanoparticles for the development of chemical sensors and biosensors

**DOI:** 10.1007/s00604-022-05536-7

**Published:** 2022-10-28

**Authors:** George Z. Tsogas, Athanasios G. Vlessidis, Dimosthenis L. Giokas

**Affiliations:** 1 Laboratory of Analytical Chemistry, Department of Chemistry, Faculty of Sciences, Aristotle University of Thessaloniki, 54124 Thessaloniki, Greece; 2 Laboratory of Analytical Chemistry, Department of Chemistry, University of Ioannina, 45110 Ioannina, Greece

**Keywords:** Analyte-mediated nanoparticle formation, Seeding growth, Enzyme-induced growth, Chemical sensors and biosensors

## Abstract

The cornerstone of nanomaterial-based sensing systems is the synthesis of nanoparticles with appropriate surface functionalization that ensures their stability and determines their reactivity with organic or inorganic analytes. To accomplish these requirements, various compounds are used as additives or growth factors to regulate the properties of the synthesized nanoparticles and their reactivity with the target analytes. A different rationale is to use the target analytes as additives or growth agents to control the formation and properties of nanoparticles. The main difference is that the analyte recognition event occurs before or during the formation of nanoparticles and it is based on the reactivity of the analytes with the precursor materials of the nanoparticles (e.g., metal ions, reducing agents, and coatings). The transition from the ionic (or molecular) state of the precursor materials to ordered nanostructured assemblies is used for sensing and signal transduction for the qualitative detection and the quantitative determination of the target analytes, respectively. This review focuses on assays that are based on analyte-mediated regulation of nanoparticles’ formation and differentiate them from standard nanoparticle-based assays which rely on pre-synthesized nanoparticles. Firstly, the principles of analyte-mediated nanomaterial sensors are described and then they are discussed with emphasis on the sensing strategies, the signal transduction mechanisms, and their applications. Finally, the main advantages, as well as the limitations of this approach, are discussed and compared with assays that rely on pre-synthesized nanoparticles in order to highlight the major advances accomplished with this type of nano-sensors and elucidate challenges and opportunities for further evolving new nano-sensing strategies.

**Graphical abstract:**

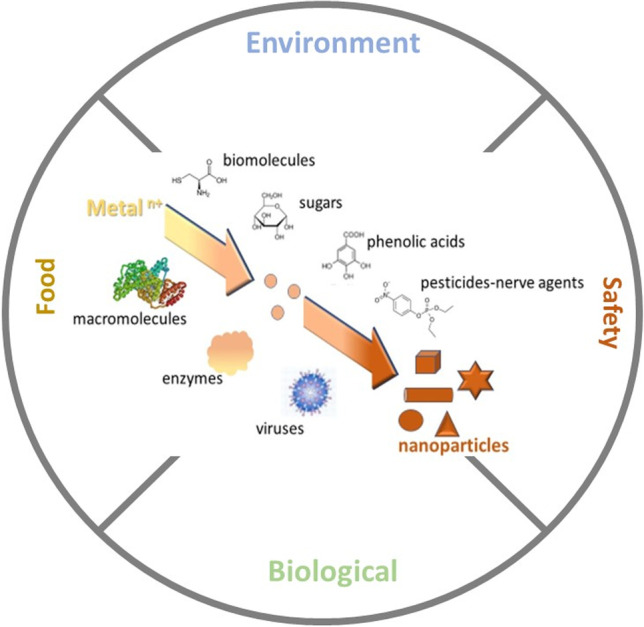

## Introduction

Nanomaterials have unique attributes, rendering them excellent scaffolds for chemical and biological sensing. Their most salient properties are their high surface-to-volume ratio, the tunable morphology (i.e., size and shape), and the versatility of their composition (metal, organic, or hybrid) that imbues nanoparticles with different reactivity and solvation properties in aqueous and organic solvents. An external stimuli event, which can modify these properties, induces changes in their physicochemical, thermal, and optoelectronic properties such as the plasmon resonance absorption, conductivity, redox behavior, thermal capacity, and catalytic activity, which are associated both qualitatively and quantitatively to the external stimulus.

In Analytical Chemistry, such external stimulus is related to the direct or indirect interaction of nanomaterials with a target analyte that may be an organic molecule, an inorganic ion, or a macromolecule (protein, enzyme, etc.). The interaction may occur either directly on the nanoparticle surface or with appropriate receptor molecules anchored on the nanomaterial surface. Following this interaction, nanomaterials undergo various physiochemical transitions that modify their properties such as a change in their aggregation state, the transformation of their morphology, or a variation in their composition (e.g., dissolution and formation of complex nano-composites). These changes and their intensity are used to detect the presence of (macro)molecules or ions and determine their concentration in a sample. Based on this strategy, enormous research effort has been devoted to the development of new analytical probes, sensors, and biosensors for the determination of a vast variety of analytes of environmental, biological, food, and industrial interest (i.e., small molecules, proteins, nucleic acids, heavy metals, and anions) [1–3]. The challenge in these methods is to prepare nanomaterials with well-defined properties that ensure a reproducible and selective response after interaction with the target analytes. Therefore, extended efforts and advanced nanotechnology skills are required to ensure the reproducible synthesis, functionalization, modification, and extensive characterization of nanomaterials before their use for analytical purposes [1–6].

A different and less represented approach is to exploit the interaction of the analytes with the precursor constituents of the nanomaterials before their assembly into organized nanostructures or during their growth into larger structures [7]. In this manner, the nanomaterials exhibit different physicochemical properties as compared to the same nanomaterials formed in the absence of the analyte. This approach alleviates the need to synthesize, characterize, and stabilize the nanoparticles before use since they are formed or grown in situ, during the application of the assay, and their properties are directly related to the presence of the analyte and its concentration in the sample. For example, during the classic synthesis of gold nanoparticles (AuNPs) by the reduction of Au ions, the color of the solution changes from yellow to red. The complexation of Au ions with thiols deters the formation of AuNPs and, consequently, the intensity of red coloration attenuates with increasing thiol concentration [8]. In contrast, the interaction of thiols with pre-formed AuNPs induces their aggregation, and the color of the solution changes from red to blue [9]. However, despite these apparent differences, studies that are based on analyte-mediated regulation of nanoparticle formation and growth have not been distinguished from nanoparticle-based assays that use pre-formed nanoparticles. Hence, these analytical methods and their principles remain “veiled” in the literature and scattered in individual articles.

In this review, we emphasize on nanomaterial-based analytical methods that rely on analyte-modulated regulation and control of nanoparticles. We provide an outline of research advances focusing on the sensing mechanisms and the concurrent detection strategies and evaluate the main characteristics and analytical merits of these methods. Not least, we try to elucidate the advantages and disadvantages of these methods and highlight fields for future research.

## Principles of analyte-mediated control of nanomaterials

The principles of sensing methods that rely on analyte-mediated control of nanomaterials are similar to those governing (a) the synthesis of nanomaterials using various additives as regulators or (b) the growth of nanomaterials using nanoparticle seeds as nucleation and autocatalytic centers.

During the synthesis of nanomaterials, except for the main building blocks (e.g. metals, carbon, and polymers), it is a common practice to use additives (e.g., thiols, amines, surfactants, etc.) to obtain a better control on the size, shape, and surface properties of the nanomaterials [10–12]. The role of these additives may be to regulate the solubility of reagents, modify the availability of active components involved in synthesis (through complexation with ions, organic molecules, or in specific sites on the nanomaterial surface), or participate in redox reactions. For example, ethylene diamines can be used to control the morphology of magnetite nanoparticles by selectively binding to the octahedral [*111*] facet without affecting their size [13]. By appropriately selecting the additives, numerous modifications have been accomplished enabling the synthesis of nanomaterials with a wide range of structures and properties [10, 12, 14].

Another popular approach for the synthesis of nanomaterials is the seeding growth method which is based on the use of very small metal particles (seeds) that serve as catalytic and nucleation centers for the growth and shaping of larger nanomaterials [15, 16]. The presence of additives in this process plays a significant role in the growth and shape of the nanomaterials and their physicochemical properties. For example, the addition of thiols during the seeding growth synthesis of gold nanoparticles has been shown to drastically inhibit the growth of nanomaterials or deter nanomaterials from re-shaping once the growth is completed [17, 18]. On the other hand, polymers or surfactants can block either the basal or the side facets of the AuNP seeds, enabling their epitaxial growth in different dimensions [15, 19, 20].

By using the target analytes as additives either before the formation or during the growth of nanomaterials, novel sensing and biosensing platforms have been developed. Depending on the function of the analytes, we classified these methods into 4 categories:

- Methods where the analytes trigger the formation of nanomaterials
- Methods where the analytes enhance the growth of nanomaterials
- Methods where the analytes inhibit the growth of nanomaterials
- Methods where the analytes affect the properties of nanomaterials

Using this categorization, we highlight key approaches and discuss the main sensing mechanisms in order to accentuate the major research advances in utilizing analyte-mediated nanomaterial sensors for the detection and determination of a variety of analytes of environmental, biological, and food interest.

## Analyte-mediated formation of nanomaterials

The interaction of the analytes with the precursor materials that constitute the building block of nanoparticles (such as metal ions, carbon materials, etc.), before the formation of nanoparticles, typically involves complexation or redox reactions that yield new reaction products with different properties. Depending on these properties, the yield and the kinetics of the nanoparticle formation reactions may be augmented or inhibited. In either case, the properties of the nanoparticles (such as size, shape, catalytic activity, etc.) when the reactions reach completion differ with increasing analyte concentration enabling their detection and determination in real samples by monitoring and comparing the optical, electrochemical, or catalytic transitions of the nanomaterial solutions in the presence and absence of the target analyte(s).

**Fig. 1:**
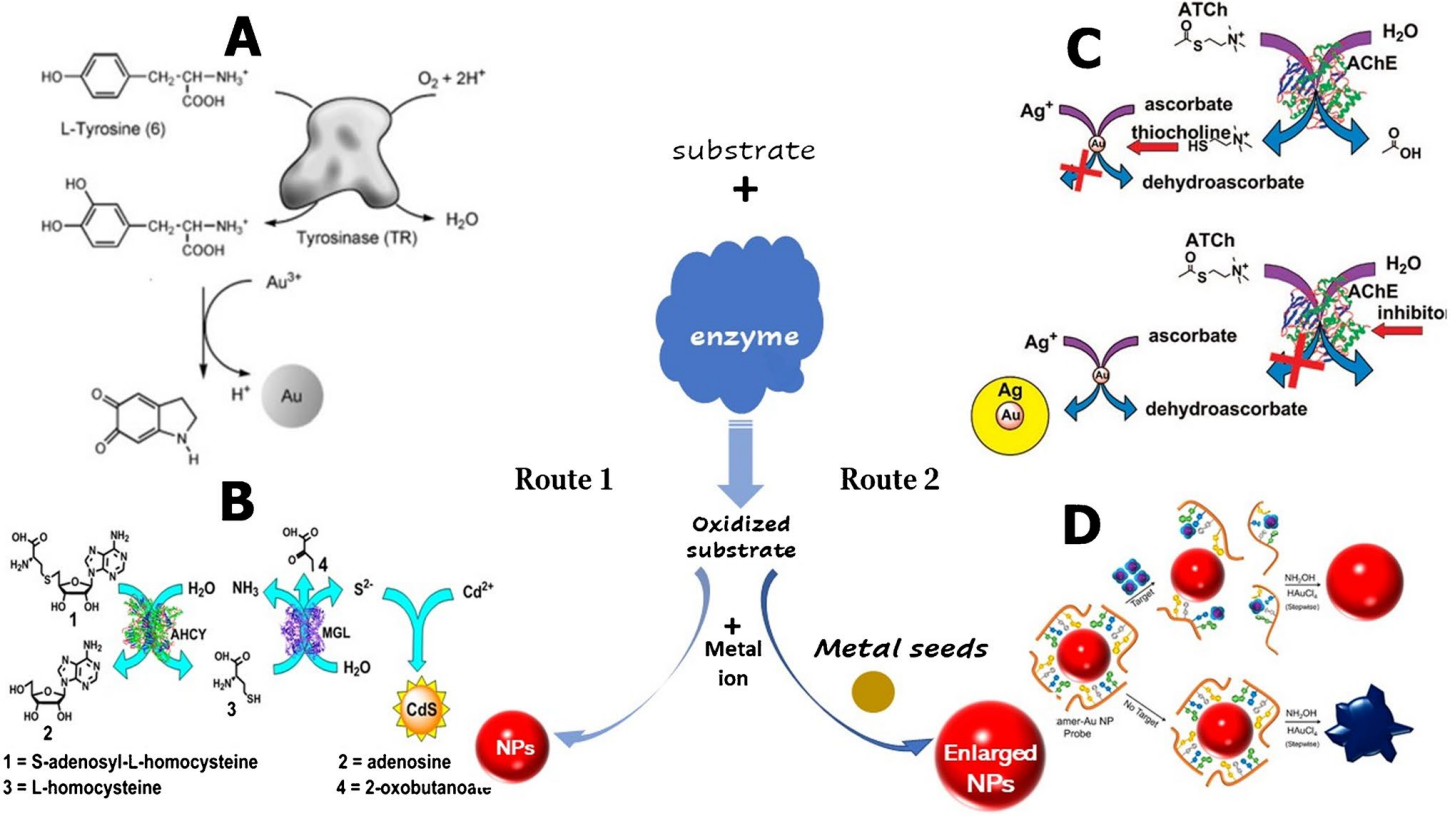
Principle of enzyme-simulated synthesis (route 1) and growth (route 2) of metallic nanoparticles. **A**) Oxidation of tyrosine by tyrosinase and reduction of gold ions by L-DOPA. Reprinted with permission from [21]. Copyright (2005) American Chemical Society. **B**) Determination of the Enzymatic Activities of MGL and AHCY using the in situ formation of CdS based on the enzymatic hydrolysis of AdoHcy by AHCY to L-homocysteine which is decomposed to S^2^^−^ by MGL. Reprinted with permission from [25]. Copyright (2012) American Chemical Society. **C**) Assay of organophosphorus nerve agents based on the inhibitory effect of thiocholine (produced from the hydrolysis of ATCh by AChE) on the growth of silver-coated-AuNP seeds. Reprinted with permission from [58]. Copyright (2009) American Chemical Society. **D**) Determination of OTA, cocaine, and 17*β*-estradiol by desorption of aptamer strands from the AuNP surface and inhibition of AuNP growth. Reprinted with permission from [60]. Copyright (2015) American Chemical Society. More details are described in the text

A classic paradigm of analyte-mediated, nanomaterial-based assays is the enzyme-simulated synthesis of metallic nanoparticles from their precursor metal ions [7]. The principle of these assays relies on the biocatalyzed oxidation of substrates towards the formation of substances that act as reducing agents of metal ions (Fig. 1-route A). The formation of metallic nanoparticles by these reactions is directly related to the concentration of the substrate and is monitored by the corresponding changes in the optical properties of the solutions due to the formation of metallic nanoparticles.

The principles of enzymatic oxidation were used for probing tyrosinase activity by exploiting the biocatalyzed hydroxylation of tyrosine to levodopa (L-DOPA) by tyrosinase (Fig. 1A) [21]. L-DOPA (a precursor of the neurotransmitter dopamine) reduces Au to AuNPs to form small AuNPs which grow in size with increasing L-DOPA concentrations [21, 22]. The absorbance increases linearly from 2.5 × 10^−6^ to 2 × 10^−5^ M of L-DOPA, which means that AuCl_4_^−^ should be present in an excess of 10–80 times that of L-DOPA to generate enough AuNPs that can produce a measurable absorbance signal. By this approach, ~ 10 units of tyrosinase (or 100 µg L^−1^) could be determined in real samples. Similarly, the determination of tyramine was accomplished by recording the molecular absorption spectra of AuNPs, formed 25 min after the enzymatic reaction of tyramine with tyramine oxidase in the presence of Au ions [23]. A novelty in this system is that Au(III) not only acts as an independent reagent that is reduced from the products of the enzymatic reaction but is also participates in the enzymatic reaction. However, other biogenic amines were found to interfere with the analysis, due to their ability to form coordination complexes with Au(III) ions, which necessitated sample pre-treatment to alleviate their interference. Despite these challenges, the method was successfully applied to the determination of tyramine in cheese samples at concentrations as low as 2.9 µM with good accuracy and precision as compared to a standard analytical protocol.

Another classic enzymatic reaction is the alkaline phosphatase (ALP)-catalyzed hydrolysis of 4-aminophenol phosphate (APP) to the reducing agent p-aminophenol which was used to trigger the in situ formation of silicon nanoparticles (Si CNPs) from N-[3-(trimethoxysilyl)propyl]ethylenediamine (DAMO), as a silicon precursor [24]. The Si CNPs were produced within 20 min at 70 °C and were water-dispersible, strongly photo-, salt-, and pH-stable yielding both an orange-red coloration with a maximum absorbance peak at 480 nm and yellow-green fluorescence at 524 nm under the irradiation of UV light (365 nm). Based on this mechanism, a dual-readout enzyme-linked immunosorbent assay (ELISA) was established for the determination of prostate-specific antigen (PSA). The ELISA components, i.e., the capture antibody, PSA, the primary antibody, and an ALP-secondary antibody conjugate, were immobilized on a 96-well plate via specific antigen–antibody immunoreactions. Upon the addition of 4-AAP and DAMO, at strong alkaline conditions (pH 9.8) which favor the ALP-triggered dephosphorylation reaction and in the presence of 20 µM Mg^2+^ to activate the ALP and increase its stability against autolysis, the Si CNPs were produced providing a fluorimetric and colorimetric signal which increased linearly with PSA concentration from 0.02 to 20 ng mL^−1^ and exhibited a detection limit of 0.0096 ng mL^−1^ [24].

Pavlov and co-workers used an unconventional route for the in situ synthesis of fluorescent CdS quantum dots (QDs) in order to develop assays for specialized analytes such as methionine γ-lyase (MGL) and S-adenosyl-L-homocysteine hydrolase (AHCY) [25]. The principle of the MGL assay relies on its ability to catalyze the decomposition of homocysteine (HCy) to H_2_S which generates fluorescent CdS in the presence of Cd^2+^ ions. The AHCY assay uses the same sensing strategy, but it is precedent by the enzymatic hydrolysis of S-adenosyl-L-homocysteine (AdoHcy) to HCy which is then decomposed by MGL (Fig. 1B). Based on these reactions, the developed methods demonstrated 200-fold better sensitivity than the conventional chromogenic assay for MGL and a detection limit of 63 µg L^−1^ for AHCY. Moreover, the sensing strategy could be used for screening AHCY inhibitors such as adenine derivatives [27]. An advantage that emerged from this in situ, analyte-mediated synthesis of QDs, is the lower background signals and the high sensitivity compared to conventional assays that employ pre-synthesized semiconductor QDs which exhibit high background signals due to unavoidable adsorption of decorated QDs on surfaces or insufficient quenching of a donor couple. However, high concentrations of Cd^2+^ ions (7.5 mM) were necessary in order to obtain the fluorescence signal, which necessitates careful handling due to its high toxicity.

Besides enzymatic methods, non-enzymatic assays have been also developed. Some of the most characteristic examples concerned the determination of antioxidant activity. These assays relied on the reduction of metal ions by phenolic acids and polyphenols to produce metal nanoparticles. The presence of a cationic surfactant, citrate ions, and a mildly alkaline pH (8.0) were necessary to stimulate the formation and growth of nanoparticles and ensure their stability. Noble metals such as Au and Ag were mostly used for these applications due to their high redox potential and intense surface plasmon resonance which induced intense colorimetric and spectral transitions in the visible region of the electromagnetic spectrum [26, 27]. The intensity of these transitions was found to depend on the *n*-electron reductant hydroxyl groups of antioxidants, which means that it was proportional to the total number of —OH groups [28, 29]. The presence of a methoxy moiety (for example, in vanillic, coumaric acid, and ferulic acids) was also found to contribute positively to the reducing strength [29]. These observations were similar to those made in other antioxidant assays [30]. Therefore, nanoparticle-based antioxidant assays, in analogy to standard antioxidant assays, did not exhibit selectivity for specific species, and they were suitable for the determination of the total antioxidant capacity that derived from the cumulative action of phenolic acids and other sample components. This attribute rendered these methods suitable for both quantitative analysis (by expressing antioxidant strength as equivalent to a standard compound such as gallic acid, caffeic acid, and Trolox) as well as for qualitative analysis by comparing the signal intensity among different samples even by the naked eye [28, 29, 31]. Another important advantage is that antioxidant-mediated nanoparticle assays exhibited a statistically significant correlation to standard assays (e.g., Folin and Ciocalteu, CUPRAC, and FRAP) enabling data comparison with other methods.

A similar reaction and sensing mechanism was used for the determination of reducing sugars (glucose, mannose, fructose, sorbitol, xylitol, etc.) in food and biological samples using their reducing action on Au or Ag ions [32–37]. The determination of reducing sugars was performed at alkaline conditions (NaOH 0.1 M) to ionize sugars and exploit the high reducing ability of their unprotonated hydroxyl groups. The presence of cationic surfactants (cetyl-trimethyl ammonium halides) as stabilizers of AuNPs was necessary since, in the presence of reducing sugars only, AuNPs were not stable and their synthesis was not reproducible [35, 36]. Since sugars are not ionized at pH < 8, selectivity against polyphenols that exhibit strong reducing activity even at lower pH could be accomplished enabling the simultaneous determination of polyphenols and reducing sugars in the same sample [33].

An interesting mechanism involving the reduction of Ag^+^ ions by carbon dots (C-dots) and the formation of silver nanoparticles (AgNPs) was used to develop a new colorimetric method for biothiols based on the AgNPs plasmon absorption. The amine and phenol hydroxyl functional groups on the C-dots first complexed and then reduced free Ag^+^ ions, towards the formation of uniformly distributed AgNPs of approximately 18 nm in size. When biothiols were present in the solution, they first formed coordination complexes with Ag^+^ ions inhibiting their direct adsorption and reduction by C-dots. The Ag-thiol complexes were then gradually reduced by C-dots leading to the formation of cysteine capped AgNPs with a wide and inhomogeneous size distribution and the absorbance increased with thiol concentration [38, 39]. This approach exhibited high sensitivity for the determination of ultra-trace amounts of biothiols with detection limits of 1.5, 2.6, and 1.2 nM for cysteine (Cys), homocysteine (Hcy), and glutathione (GSH), respectively.

**Fig. 2:**
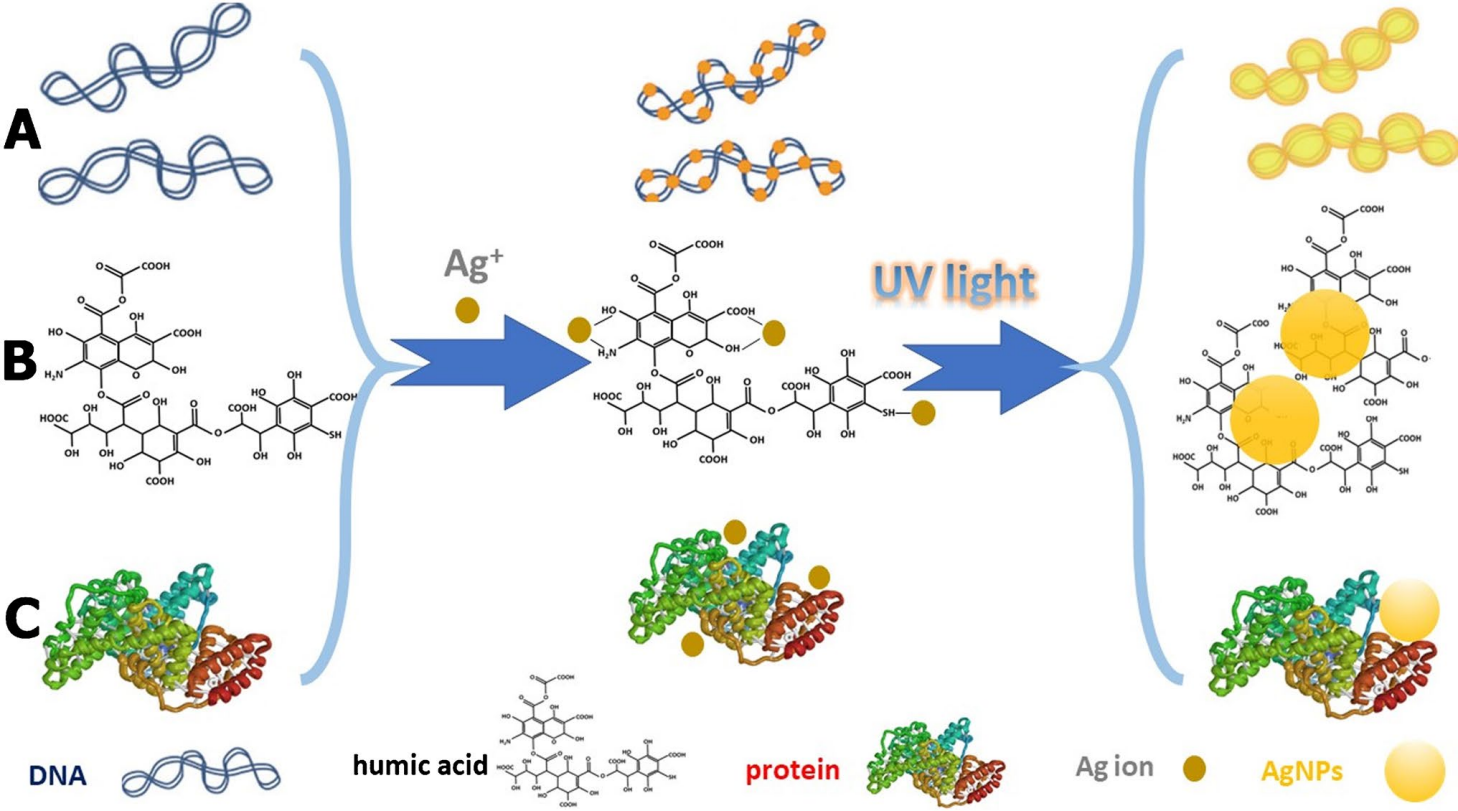
Principle of analyte-mediated, photochemically assisted synthesis of nanomaterials. **A**) Detection of bacterial genomic DNA of pathogenic bacteria based on Ag.^+^ ion reduction promoted by photoirradiation of DNA. Republished with permission of the Royal Society of Chemistry from [41], **B**) DOM-mediated photo-reduction of Ag^+^ ions to AgNPs. **C**) Discrimination of proteins by protein-templated photosynthesis of AgNPs. Adapted by permission from the Springer Nature [42]

The photochemically assisted synthesis of nano-scale materials has been also employed for developing new analytical probes by exploiting the target analytes as nanoparticle growth agents (Fig. 2). All analyte-mediated, photochemically assisted assays were performed under UV light irradiation using silver ions and macromolecules as the target analytes. In the absence of the analyte, the photoreduction of silver ions led to the formation of elemental silver or silver oxide which did not induce a change in the color of the solution (except for the formation of dark gray aggregates at high silver concentrations) [40, 41]. When the target analyte was present, it complexed with silver ions and led to the formation of AgNPs with colorimetric and absorbance properties related to the concentration of the analyte and its chemical structure [40–42].

The photo-induced silver ion (Ag^+^) reduction to AgNPs around DNA bases was found to be a useful route for the direct detection of bacterial genomic DNA without the need for PCR amplification. The method proceeded with the initial complexation of Ag^+^ ions by DNA followed by exposure of the complex to UV light (254 nm) (Fig. 2A). At least 1.5 mM AgNO_3_ was required to ensure the formation and growth of an adequate amount of AgNPs on DNA that could produce a yellow-colored suspension, with a surface plasmon resonance peak at 420 nm [41]. Another unique feature of this light-mediated reaction system is that sensitivity increased with the length of the target DNA due to the presence of more nucleobases which increased the reducing power of the target DNA while also increased the specific Ag^+^-binding sites of the polynucleotide. Due to this feature and the fact that the size of bacterial genomic DNA is typically in the range of 0.6 Mbp to over 10Mbp, the detection limit of the assay was 67 pg mL^−1^, a value that was lower or comparable to those of previously reported colorimetric DNA detection methods.

Based on the same principle, Pu et al. developed a sensor array for the determination and discrimination of proteins using the unique effect of each protein in the photoreduction of AgCl to AgNPs [42] (Fig. 2C). The different structures and properties of each protein led to the formation of AgNPs with distinct peak shapes, positions, and intensities of their SPR absorption spectra. Simple chemometric tools such as principal component analysis could effectively discriminate among 10 proteins with different molecular weights and isoelectric points.

Another example of analyte-driven formation of nanoscale materials assisted by UV light is the reduction of silver ion-dissolved organic matter (DOM), complexes, mainly humic and fulvic acids. The mechanism of AgNP formation involved a two-step process starting with the complexation of Ag^+^ by DOM and followed by the ligand-to-metal charge transfer upon light exposure [40]. The absorbance intensity of the solution increased with DOM concentration in the sample due to the formation of DOM-coated AgNPs (Fig. 2B). Aromatic-based DOM was more effective in reducing Ag^+^ to colloidal silver than aliphatic-dominated DOM, while the concentration of DOM was also found to affect the particle size distribution of the formed AgNPs. Lower DOM concentrations produced AgNPs of larger size distribution, as evidenced by the board absorbance spectra, an observation that was attributed to the insufficient stabilization of AgNPs. In addition, the water matrix was found to affect the formation of AgNPs; therefore, matrix-matched calibration was used to determine DOM concentrations (as the sum of both humic and fulvic acids) at environmentally relevant concentration levels (0.5–5 mg L^−1^) and detection limits as low as 0.38 mg L^−1^.

## Methods based on analyte-mediated growth of nanomaterials

The analyte-mediated seeding growth of nanoparticles is based on two mechanisms that may occur separately, simultaneously, or sequentially. The first involves the interaction of the analytes with the precursor materials, restricting or enhancing their contribution to the growth of nanoparticle seeds. The second mechanism involves the interaction of the analytes with the nanoparticle seeds affecting their catalytic activity and growth. Both mechanisms influence the growth of nanoparticles and consequently their physicochemical properties in a manner analogous to the analyte concentration in the sample (Fig. 1-route 2).

The enzyme-stimulated growth of nanoparticles based on the catalytic deposition of metals on nanoparticle seeds is the first and most popular strategy that utilized the growth of nanoparticles for analytical purposes. The first study was published by the Willner group and employed the catalytic growth of AuNPs by nicotinamide adenine dinucleotide phosphate (NAD(P)H) cofactors [43]. More specifically, they described the determination of lactate in the presence of the NAD^+^-dependent lactate dehydrogenase (LDH) which proceeded by the reduction of AuCl_4_^−^ to Au (I) from nicotinamide adenine dinucleotide (NADH) and then by the catalyzed reduction of Au(I) by the AuNP seeds [43].

Another characteristic example is the enzymatic determination of glucose based on its oxidation by glucose oxidase to generate H_2_O_2_. The latter reduced AuCl_4_^−^, catalyzed by AuNP seeds, resulting in the enlargement of AuNP seeds and enhanced the absorbance of the solution [44]. Xiong et al. optimized the reaction conditions of this system and evaluated the assay in real samples from both healthy and diabetic individuals demonstrating its advantages in clinical testing [45]. Using this principle, a more sophisticated method for the determination of PSA as a prostate cancer biomarker was developed using gold nanostars modified with polyclonal anti-PSA and anti-mouse immunoglobulin G (IgG) conjugated to glucose oxidase (GOx) as a label. GOx produced H_2_O_2_ which reduced Ag^+^ ions to grow a silver coating around plasmonic gold nanostars (60 nm) favoring their growth with a conformal silver coating [46]. The method exhibited outstanding sensitivity for PSA, down to 0.04 aM in whole serum. The use of spherical AuNPs of 5 nm size, instead of gold nanostars, exhibited lower sensitivity for PSA determination (LOD = 93 aM) [47] but still several orders of magnitude (> 4) to that of commercial ELISA (6.3 pM).

One of the most widely adopted biocatalytic routes for the growth of metal nanoparticles is the hydrolysis of p-aminophenyl phosphate (p-APP) from ALP to p-aminophenol. The enzymatically generated p-aminophenol reduced Ag^+^ ions, catalyzed by AuNP seeds, causing their enlargement. This route was initially developed as a method to deposit Ag nanowires on AuNP decorated surfaces by dip-pen nanolithography [48] but found several applications in the development of analytical probes and biosensors. In the first analytical method to use this strategy, ALP was used to hydrolyze thiophosphate to orthophosphate and H_2_S. The latter reacted immediately with Cd^2+^ cations to produce fluorescent CdS QDs. Therefore, in the presence of a fixed concentration of thiophosphate, ALP activity could be determined at concentrations up to 57 ng mL^−1^ with a detection limit of 8 ng mL^−1^ [49].

A similar reaction route using 4-APP as substrate was used as a signal amplification strategy in immunoassays [50–54] and cascade hybridization chain reactions [55, 56] affording a significant improvement in the sensitivity. All of these assays relied on staining AuNPs with silver, which causes large spectral changes since the SPR of AuNPs and AgNPs differ by almost 200 nm. The analytical procedure first followed the standard procedure used in sandwich-type immunoassays, where antibodies are used to capture the target antigens and sandwiched with a detection antibody conjugated with ALP. Then, 4-APP as substrate as well as AuNPs and Ag^+^ ions as a signal reporter and amplification probes, respectively, were added. 4-APP was hydrolyzed by ALP to generate 4-aminophenol that reduced silver ions to metallic silver. The latter deposited on AuNPs generating a shift in the absorbance spectra and a change in the color of the solution. In contrast, in the absence of ALP, no color was generated.

The selection of the appropriate AuNP seed morphology in these enzyme-mediated biometallization reactions is critical in improving the sensitivity of the assays. Gold nanorods (AuNRs) have been the most widely used nanoparticles, and their use as signal amplification reporters has been described for the development of PSA rabbit and immunoglobulin G [53] immunoassays using the ALP/4APP/Ag reaction system [54] as well as for the detection of *Escherichia coli* based on the hydrolysis of the substrate p-aminophenyl β-D-galactopyranoside by β-Gal to *p*-aminophenol [52]. Other AuNPs such as spherical AuNPs were used to detect Avian influenza virus (H9N2) particles [51] and for the sequence-specific DNA detection (after hybridization chain reaction) accomplishing a noticeable improvement in sensitivity compared to standard methods [56]. Gold nanobipyramids (Au NBPs) as reporter probes of influenza (H5N1) virus in sandwich immunoassay [50] and gold nanostars (Au NS) combined with hybridization chain reaction amplification for the determination of DNA were also reported [55]. All of these assays rely on gold–silver staining which induces large spectral changes since the SPR of AuNPs and AgNPs differs by almost 200 nm. Only recently, the direct growth of AgNPs by Ag ions was reported for the determination of ALP activity [57] over a wide dynamic linear range of 0.5–225 U L^−1^ (LOD = 0.24 U L^−1^) compared to the Ag/AuNP probes.

A paradigm shift in assays which rely on analyte-mediated growth of nanoparticles is the observation that some enzymes or the products of their hydrolysis could inhibit the growth of nanoparticles, instead of enhancing it. In this occasion, enzyme inhibitors were used to enhance the formation of nanoparticles by blocking the inhibitory action of enzymes or their hydrolysis products. This concept was put forth for manufacturing a simple colorimetric assay for determining nerve gases as AChE inhibitors (Fig. 1C) [58]. In the absence of nerve agents, the enzymatically produced thiocholine, bound to the surface of the AuNP seeds, and hindered the deposition of silver (Ag^0^) which was reduced from AgNO_3_ (Ag^+^) with ascorbic acid. Nerve agents could decrease the formation of thiocholine; therefore, a silver coating gradually built on the surface of AuNP seeds and caused their enlargement.

Similarly, the determination of tyrosinase (TYRase) concentration in blood serum samples by monitoring the formation of core–shell Au@Ag bimetallic nanoparticles from AuNP seeds was reported [59]. In the absence of TYRase, core–shell Au@Ag bimetallic nanoparticles could be formed by the reduction of Ag^+^ ions on AuNP seeds with kojic acid as a reducing agent. In the presence of TYRase, kojic acid could complex with the di-copper active site of TYRase; hence, the concentration of free kojic acid in the solution decreased. As a result, the in situ metallization of Ag^+^ on AuNPs was inhibited, and the color of the solution gradually shifted from yellow (in the absence of TYRase) to pink (in the presence of TYRase). The detection limit of the method was as low as 0.019 U mL^−1^, but common biomolecules and inorganic electrolytes were found to interfere with the quantification, dictating appropriate sample pre-treatment steps (such as dialysis) before analysis.

Except for assays that rely on the enzyme-analyte interactions for the growth of metal nanoparticles, several non-enzymatic reactions have been also reported. The main difference from enzymatic reactions is that the reducing agent is not produced in situ from a reaction that involves the contribution of the target analyte, but it is added into the solution as a reactant, along with the other reagents, to stimulate AuNPs growth. Such non-enzymatic seeding growth reactions combined with aptamer–target recognition were utilized to determine small molecules by regulating the growth of aptamer-functionalized AuNPs in the presence of AuCl_4_^−^ as a growth factor and NH_2_OH as a reducing agent of Au ions [60, 61]. In the absence of target analytes, the aptamers protected the AuNPs, while in the presence of the target molecules, the highly specific aptamer–target interactions triggered the desorption of the aptamers from the AuNPs surface. The removal of the aptamer protective coating enabled the deposition and reduction of AuCl_4_^−^ on the AuNPs surface thus causing their enlargement (Fig. 1D). Due to the use of aptamers, these methods exhibited high specificity, while the use of AuNPs offered high sensitivity for the determination of various small molecules such as ochratoxin A (OTA) in wine samples, cocaine in synthetic urine, 17β-estradiol (estradiol) in saliva, and cholic acid.

Electron donor molecules such as sugars and phenolic compounds or polyphenols could also stimulate the catalytic deposition of metal ions on metal nanoparticles, and this property was used for the development of assays that measure the reducing (antioxidant) strength of the samples. The silver mirror reaction generated from the reduction of Ag(NH_3_)_2_OH by glucose and the subsequent deposition of metallic silver on AuNPs enabled the development of a glucose probe in serum samples [62]. However, despite the simple operation and the fast analysis time the method was prone to interferences from other reducing agents and proteins, commonly found in biological samples, necessitating specific sample pre-treatment steps before application.

The determination of the total reducing strength was conveniently accomplished by the seed-mediated growth of noble metal nanoparticles in antioxidant-rich food matrices [63] by immobilizing AuNP seeds on cysteamine-coated Au electrodes and immersing the electrodes in a growth solution composed of AuCl_4_^−^ and flavonoids as mild reductants. Flavonoids caused the growth of the AuNP seeds to AuNPs affecting the electrochemical response in a manner analogous to flavonoid concentration in the sample. This method was used for the determination of the concentration of flavonoids in different extracts from natural plants with very low detection limits that reached the µM levels. In a more generic application, Apak et al. developed a sensitive colorimetric method for the determination of total polyphenols based on their ability to reduce Ag^+^ ions in the presence of citrate-stabilized AgNP seeds [64]. The deposition of Ag^+^ ions on the surface of AgNP seeds caused their enlargement, increasing the SPR absorption band of AgNPs at 423 nm linearly with polyphenols concentration. The method was applied to the determination of the total antioxidant activity of plant extracts, and the results correlated well with standard spectrophotometric assays such as the CUPRAC assay [74].

## Assays relying on analyte-mediated inhibition of nanoparticles growth

**Fig. 3:**
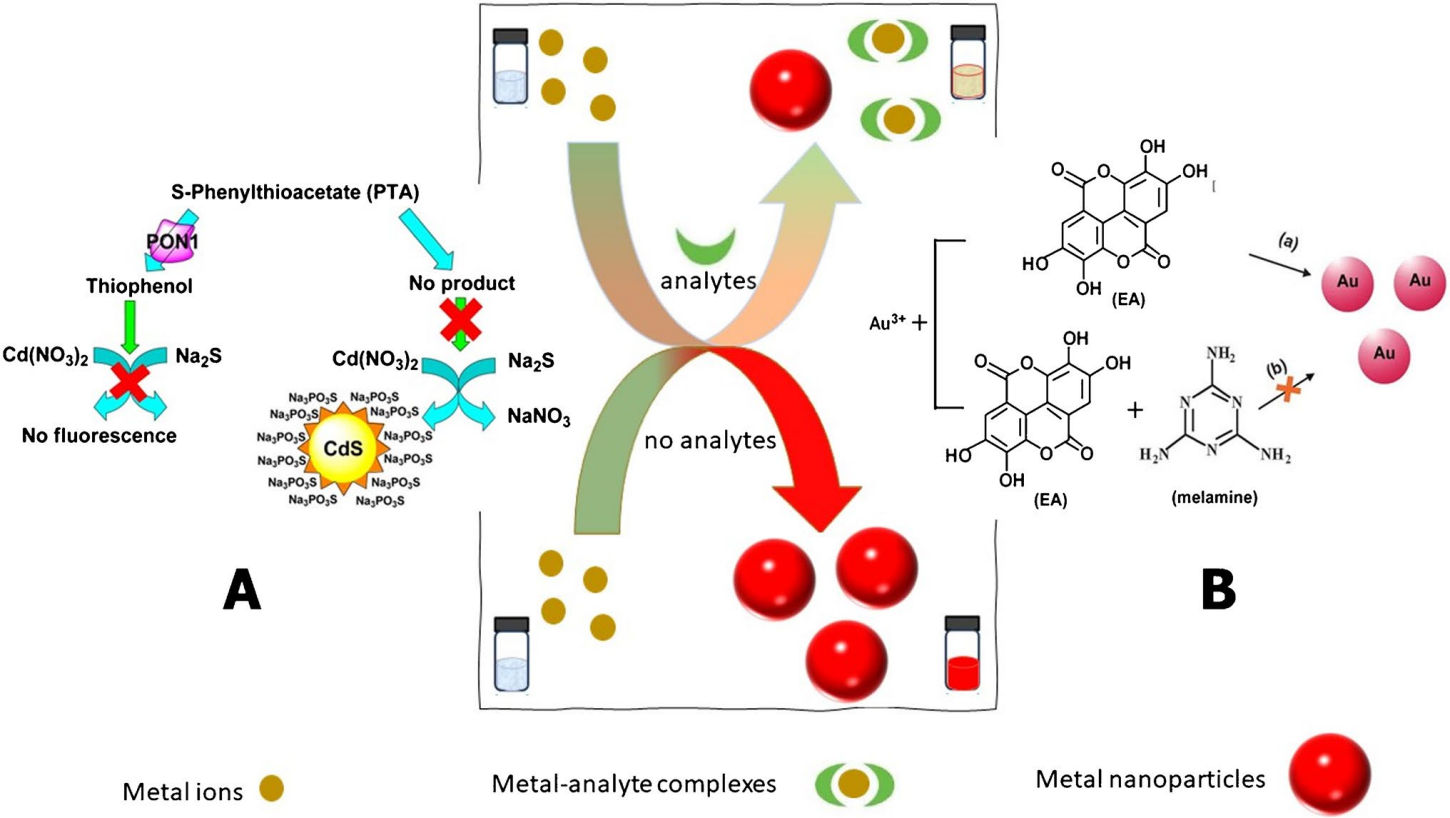
Principle of analyte-mediated inhibition of nanomaterials formation. **A**) Detection of PON1 activity by the enzymatic modulation of the formation of fluorescent CdS QDs. Thiophenol formed by the hydrolysis of PTA with PON1 inhibits the generation of fluorescent CdS from Cd^2+^ and S.^2−^ ions in the presence of sodium thiophosphate (Na_3_PO_3_S) as a stabilizer. Reprinted with permission from [69]. Copyright (2009) American Chemical Society. **B**) Determination of melamine based on the interruption of the synthesis of gold nanoparticles formed by the reducing action of ellagic acid in Au ions. Republished with permission of the Royal Society of Chemistry from [72]

Except for the capability of analytes to trigger the formation or enhance the growth of nanomaterials, several methods have exploited the ability of analytes to interfere or inhibit the growth of nanomaterials. In these assays, the precursor materials, necessary for the formation of the nanoparticles, are used in adequate excess in order to ensure the formation and the growth of nanoparticles and the generation of an analytical signal. The target analytes either interfere with the formation of nanoparticles and reduce the amount of nanoparticles that are produced or inhibit their growth concurrently causing a decrease in the analytical signal that is analogous to the concentration of the target analyte in the sample (Fig. 3). Complete inhibition of nanoparticles formation or growth may be accomplished at a very large concentration of the analyte.

One of the first analyte-inhibition assays that was developed concerned the determination of AChE enzyme inhibitors, such as pesticides, by exploiting their ability to reduce the hydrolysis of acetylthiocholine (ATCh) by acetylcholinesterase (AChE) to produce thiocholine (TCh) which is a mild reducing agent. TCh could reduce AuCl_4_^−^ ions in order to accomplish the deposition of gold on the AuNP seeds (dispersed in solution or deposited on glass surfaces). Therefore, the more TCh in the solution, the more AuNP seeds grew in size and consequently gave rise to intensified, broad, and blue-shifted absorbance bands [65]. AChE inhibitors interfered with the production of TCh, impairing the growth of AuNP seeds in a manner proportional to their concentration in the sample. By this approach, AChE inhibitors such as organophosphorus pesticides could be optically detected at concentrations as low as 0.6 nM.

Further research studies employing the same principle exploited the direct reduction of gold ions to AuNPs without the addition of AuNP seeds by immobilizing AChE on the surface of chitosan-coated gold electrodes [66] or glass slides [67]. The AChE-modified surfaces were immersed into the sample solution, containing AChE-inhibitor pesticides as analytes, ATCh, and AuCl_4_^−^ ions. AChE triggered the hydrolysis of ATCh to TCh which in turn reduced AuCl_4_^−^ to AuNPs. AChE-inhibitor pesticides decreased the hydrolysis of ATCh to TCh proportionally to their concentration in the sample and consequently reduced the formation of AuNPs. Analysis was carried out either electrochemically or optically. In the first occasion, quantification was performed by monitoring the increase (∼50%) in peak currents due to the significant increase in the electron transfer rate across the electrode interface caused by the deposition of AuNPs, within 10 min upon addition of the sample (since at larger incubation times AuNPs congregated to packed clusters resulting in an increase of the electrode interface resistance that block the electron transfer) [66]. In the other method, the changes in the orientation of liquid crystals were optically recorded after 1.5 h of incubation which was necessary to ensure an adequate change of the surface topology of the liquid crystal cell and a homeotropic-to-tiled transition of the liquid crystal molecules [67]. Using these inhibition reactions, the presence of OP pesticides could be determined optically at concentrations lower than 0.3 nM and electrochemically at concentrations as low as 0.1 nM.

Another enzymatic assay for the determination of serum paraoxonase (PONI1) arylesterase activity was based on the inhibition of the formation of fluorescent CdS QDs. PON1 hydrolyzed S-phenyl thioacetate (PTA) to form thiophenol which inhibited the generation of fluorescent CdS from Cd^2+^ and S^2−^ (Fig. 3A) [68]. As a result, the fluorescence of the solution was quenched proportionally to the concentration of thiophenol. Quenching was attributed to the blocking of the CdS surface by thiophenol, inhibiting their growth. However, due to the high pH of the reaction (pH 8) and the high complex formation constants of thiols with Cd^2+^, it is also possible that thiophenol may have complexed Cd^2+^ ions inhibiting the formation of CdS [69, 70]. The addition of sodium thiophosphate (Na_3_PO_3_S) was necessary to stabilize the QDs and ensure a stable fluorescence response. The lowest PON1 activity that could be detected by this analytical system was 0.625 mU mL^−1^ which is almost 15 times lower compared to that of the conventional spectrophotometric assay.

Except for enzymatic methods, analyte-mediated, nanoparticle inhibition assays were developed for the determination of melamine by exploiting its ability to form hydrogen bonds with the carboxyl and -OH groups of reducing compounds. The sensing mechanism was based on the use of ellagic acid or 3,5-dihydroxybenzoic acid as reducing agents of AuCl_4_^−^ ions to AuNPs, yielding a red to purple coloration [71, 72]. When melamine was present, it formed hydrogen bonds with the reducing agents, weakening their reducing strength and consequently inhibiting the formation of AuNPs (Fig. 3B). A blue shift in the absorbance spectra was gradually observed with increasing melamine concentration (as compared to the bank sample in the absence of melamine) until a yellowish coloration appeared which is characteristic of the AuCl_4_^−^ ions. Both of these methods achieved the determination of melamine at the low nanomolar concentration levels.

The inhibitory effect of analytes on the photochemically assisted reduction of noble metals is another process that was utilized for the development of nanoparticle inhibition assays. In contrast to silver-analyte complexes, which are favorably reduced to AgNPs under the influence of UV light, as discussed above [40–42], the complexation of gold ions inhibits their photoreduction by UV light, while free gold ions are effectively reduced to AuNPs when an electron donor (such as citrate anions) are present in the solution. As an example, the inhibitory effect of thiols on the photo-induced formation of AuNPs was used to determine biothiols (such as cysteine, homocysteine, and glutathione) in biofluids. The principle of detection relies on the fact that UV light can trigger the photo-reduction of AuCl_4_ ions to AuNPs in the presence of a mild reducing agent (such as the amino acid threonine). When biothiols were present in the sample, the formation of AuNPs was obstructed. In this manner, the red color of the AuNP solution gradually faded, becoming colorless at high biothiol concentrations [8]. Consequently, the absorption intensity of AuNPs at 530 nm was reduced linearity with biothiol concentrations up to 300 µM and logarithmically at higher concentrations. Based on this phenomenon, the determination of total biothiols in serum samples was reported with detection limits that ranged from 46 to 76 µM (based on a signal-to-noise ratio (S/N) of 3) depending on the biothiol species.

Beyond the inhibition in the photochemically assisted formation of AuNPs, Kostara et al. [73] observed that a large variety of sulfur-containing compounds including thiols, thioesters, disulfides, thiophosphates, metal–sulfur bonds, and inorganic sulfur can also slow down the photoreduction kinetics of Au ions to AuNPs (in the presence of citrate anions as sensitizer). The color of the sample solutions changed from red (in the absence of analytes) to purple (in the presence of analytes), but the time required for the process to reach completion increased proportionally to analyte concentration in the sample. The different reaction kinetics with increasing analyte concentrations was used to develop instrumentation-free, time-based assays for various sulfur-containing compounds such as dithiocarbamate and organophosphorus pesticides, biothiols, pharmaceutically active compounds, and sulfides in different samples (natural waters and wastewater, biological fluids, and prescription drugs) with good linearity and satisfactory sensitivity [73]. By comparing the reaction kinetics, some selectivity among compounds belonging to the same category could be also achieved.

The inhibitory effect of biothiols in the formation of AgCl (nano)crystals and the concomitant influence on the photosensitivity of the suspension is another example of a non-enzymatic method where the analyte could inhibit the formation and growth of nanomaterials. In the absence of biothiols, AgCl crystals could rapidly form upon mixing AgNO_3_ and NaCl and grow in size with increasing chloride concentrations due to Ostwald’s and coalescence ripening [74]. The AgCl suspension exhibited high photosensitivity upon exposure to UV light and turned dark due to the formation of metallic silver. On the other hand, when biothiols were present in the solution, they formed complexes with Ag^+^ and inhibited the formation and growth of AgCl crystals. Consequently, the photosensitivity of the AgCl suspension was reduced due to the lower amount of AgCl in the suspension, and the color of the UV-irradiated AgCl suspension became brighter with increasing biothiol concentration. The difference in the incident light absorbed by the AgCl suspension in the presence and absence of thiols was related to the concentration of biothiols in the sample solution [74]. This approach was successfully utilized for the determination of biothiols in urine and blood plasma with detection limits as low as 10 µM. Moreover, the method did not respond to oxidized biothiols (e.g., cystine) enabling the differentiation between reduced/oxidized thiols, which is an indicator of cellular health status.

## Analyte-mediated regulation of nanoparticle properties

The interaction of analytes with the precursor materials or nanoparticle seeds may not only affect the formation and growth of nanoparticles but also their properties and morphology. In these cases, the analytical signal is related both to the change in the properties of the nanoparticles and their formation or growth.

**Fig. 4:**
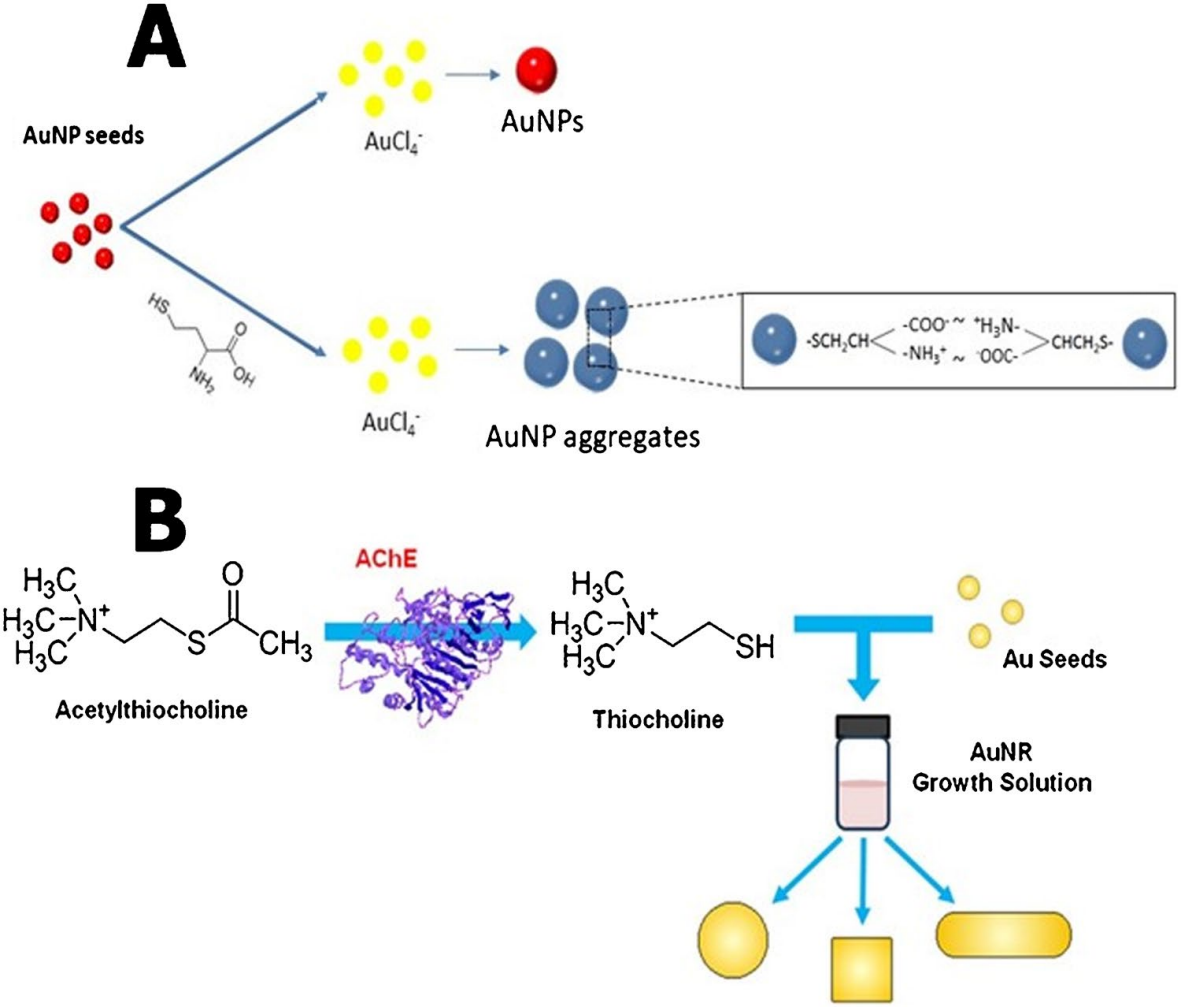
**A** Biothiol-mediated aggregation of AuNPs during their growth. Reprinted from [76] with permission from Elsevier. **B** Determination of OP nerve agents through modulation of seed-mediated gold nanorod growth by enzymatically produced thiocholine. Reprinted from [75] with permission from Elsevier

The morphological transitions induced by thiolated molecules in AuNRs were used for the development of nerve gas biosensors. Thiocholine, produced from the hydrolysis of acetylthiocholine by AChE, was used to modulate AuNR growth towards the formation of various Au nanostructures that varied from nanorods to cubes and finally to spheres, with increasing thiocholine concentration [75]. AChE inhibitors deterred the production of TCh by phosphorylating the serine hydroxyl group in the active site of AChE, and as a consequence, the intensity and the position of the surface plasmon resonance changed, due to the formation of AuNPs of different sizes and shapes, as a function of AChE inhibitor’s concentration (Fig. 4B). Based on these mechanisms, the authors developed a plasmon-assisted biosensor of OP nerve-gas agents with detection limits between 280 pM and 1 nM, depending on the incubation time of AChE with the OP nerve agents.

Recently, Akrivi et al. [76] observed that biothiols behaved both as controlling agents of AuNPs growth and aggregation in a concentration-depended manner when they were mixed with AuNP seeds before the addition of the growth solution (i.e., AuCl_4_^−^ and a mild reducing agent). At low concentrations, biothiols inhibited the growth of AuNPs by decreasing the catalytic activity of AuNP seeds, while at higher concentrations, they could cause the aggregation of AuNPs through the formation of COO-NH bonds (Fig. 4A). The color and absorbance transitions induced by these mechanisms were used to develop a colorimetric probe for the determination of glutathione in red blood cells and cysteine in blood plasma.

Pavlov and co-workers discovered that phosphate can stabilize CdS QDs and trigger their fluorescence, while no fluorescence was observed in the absence of phosphate ions. [77]. This finding was used to develop an ELISA method for the determination of anti-BSA antibodies by using ALP-conjugated antirabbit IgG and the in situ synthesis of CdS QDs by mixing Cd(NO_3_)_2_ and Na_2_S. To this system, p-nitrophenyl phosphate was added as a substrate which was hydrolyzed by ALP to produce inorganic phosphate that stabilized CdS QDs and enhanced their fluorescence [77]. The sensitivity of this method was 6.5 times higher than the standard colorimetric p-nitrophenyl phosphate method enabling the determination of the target antibody at concentrations as low as 0.4 ng mL^−1^. Other researchers used adenosine triphosphate (ATP) to stabilize CdS QDs, through interaction with its phosphate groups and enhance their fluorescence. ALP, which could hydrolyze ATP molecules to produce adenosine and phosphoric acid through enzymatic dephosphorylation, induced the precipitation of CdS QDs because neither of the products of the enzymatic hydrolysis reaction could act as a stabilizer [78]. In this manner, the fluorescence intensity decreased with increasing ALP concentration. This indirect inhibition reaction responded linearly to ALP concentrations in the range of 0.42 to 12.6 nU mL^−1^ with a LOD of 0.17 nU mL^−1^ which is lower than many previous methods.

Aminothiols could also act as stabilizers of CdS QDs during their formation and growth and enhance their fluorescence [79]. In that sense, reduced GSH could be directly determined in biological fluids by recording the increased fluorescence emitted from CdS QDs with increasing GSH concentration. Further exploitation of this observation led to the development of a multi-analyte analytical system for oxidized glutathione, NADH, and glutathione reductase (GR) activity [79]. Specifically, GR was used to break down glutathione disulfide (GSSG), to GSH using NADPH as a cofactor. When Cd^2+^ and S^2−^ were added to the solution, the produced GSH bound to the surface of the growing CdS crystals through the mercapto group, stabilizing CdS crystals and leading to the generation of fluorescence. By keeping constant the concentrations of either component, namely NADH, GSSG, or GR, either species could be determined interchangeably with very high sensitivity (i.e., ≤ 2 µM GSH, ≤ 10 µM GSSH, ≤ 2.5 µM NADH, and ≤ 5 pM GA) that surpassed many previous colorimetric and fluorescence assays.

## Discussion

To elucidate the advantages and disadvantages of analyte-mediated nanoparticle assays, compared to nanoparticle assays that rely on pre-synthesized nanomaterials, a non-exhaustive comparison of the analytical merits of each approach was performed. Table 1 summarizes the analytical parameters as well as some of the experimental and technical details of assays for the determination of the same analytes using analyte-mediated and pre-synthesized nanomaterials. The first and most obvious advantage of analyte-mediated nanoparticle assays is that they alleviate the need to synthesize, characterize, and stabilize nanoparticles with appropriate analyte-recognition properties before use. This holds true also for methods which are based on analyte-mediated growth of nanomaterials, because nanoparticle seeds are used only as nucleation centers and do not need to contain specific analyte-recognition and signal transduction mechanisms. Hence, no advanced nanotechnology skills and specialized operations, which cannot be routinely performed in ordinary analytical and biochemical laboratories, are required.

**Table 1:** Comparison of nanomaterial-based analytical methods employing analyte-mediated formation and growth of nanoparticles (in bold letters) and pre-synthesized nanoparticles

Sensing method(s)	Analyte(s)	Linear range	Detection technique	LOD	RSD (%)	Recoveries (%)	Analysis time (min)	Ref
**AChE/ATCh/Au**	Paraoxon	0–30 nM	Colorimetry	280 pM	-	-	180	[75]
**AChE/ATCh/Au**	AChE activity OP	0.015–1.5 µM 0.3–3000 nM	Colorimetry	15 µM 0.3 nM	-	-	90	[67]
**AChE/ATCh/AuNPs/Ag**	Paraoxon BW284c51	0–1.32 µM 0–1.12 nM	Colorimetry	4.0 nM 80 pM	-	-	-	[58]
MnO_2_ NPs	Tacrine	1–200 nM	Colorimetry	0.9 nM	-	-	100	[80]
PdSP@rGO	Tacrine	25–400 nM	Colorimetry	2.3 nM		93.1–103.5	40	[81]
**Au/ellagic acid**	Melamine	0.016–160 µM	Colorimetry	1.6 nM	1.6	93–106	30	[71]
**Au/3,5-dihydroxybenzoic acid**	Melamine	0.001–10 µM	Colorimetry	0.8 nM	0.33	-	50	[72]
1,4-dithiothreitol @ AuNPs	Melamine	80–600 nM	Colorimetry	24 nM	-	96–103	30	[82]
citrate@AuNPs	Melamine	0–0.9 µM	Colorimetry	33 nM	< 2	99.2–111	60	[83]
**AuNP seeding growth**	Cysteine Homocysteine Glutathione	3–300 µΜ 10–300 µΜ 5–300 µΜ	Colorimetry	1.0 µM 3.0 µM 3.0 µM	3.8–7.7 5.7–8.3 1.8–3.6	88.7–114.0	30	[76]
**AgCl /UV light**	Cysteine Homocysteine Glutathione	10–100 µΜ	Colorimetry	8.1 µM 9.0 µM 9.8 µM	6.7 7.4 8.8	92–97	20	[74]
N-CQDs-AuNPs	Cysteine	0.05–12 µM 10–100 µM	Fluorescence Colorimetry	20 nM -	< 4.4	98–104	20	[84]
VS-CDs	Cysteine Homocysteine Glutathione	5–200 µM 5–200 µM 1–200 µM	Fluorescence	0.3 µM 0.4 µM 0.3 µM	5.4	98.6–112	> 30	[85]
Aspartic acid@AuNPs	Cysteine	100–1000 µM	Colorimetry	1.0 µM	2.0	99.2–101.1	2	[86]
**Au/UV light**	Dithiocarbamate pesticides	50–400 µg L^−1^	Colorimetry/ kinetic	50 µg L^−1^	7.7	98.5–102.0	< 10	[73]
citrate@AuNPs	Dithiocarbamate pesticides	25–175 µg L^−1^	Colorimetry	11 µg L^−1^	< 10	81–94	> 10	[87]
**Au/phenolic acids**	Antioxidant activity	10 µΜ–1 mM	Colorimetry	< 1 µΜ	3.6–12.6	-	10	[29]
**AgNP seeding growth**	Antioxidant activity	~ 1–100 µM	Colorimetry	0.23 µM	< 5.2	92.3–102.7	30	[64]
**AuNP seeding growth**	Polyphenols	1–15 µM	Colorimetry	3.3 µM	< 6		17	[33]
GCN-Cu NFs	Phenolic compounds	1–100 µM	Colorimetry	0.27–0.82 µM	< 3	97.1–108.9	5	[88]
CeNPs	Antioxidant activity	0.06–2.0 mM	Colorimetry	50 µM	4	-	10	[89]
**Ag-DNA complex / UV light**	Genomic DNA	0–58.7 µg L^−1^	Colorimetry	67 µg L^−1^	-	-	20	[41]
oligonucleotide@AuNPs	Genomic DNA	27–430 ng	Colorimetry	54 ng			20	[90]
**In situ CdS QDs formation**	ALP	0–50 U L^−1^	Fluorescence	0.5 U L^−1^	-	-	90	[77]
**In situ SiNP formation**	ALP	0.1–6.0 U L^−1^ 0.1–8.0 U L^−1^	Fluorescence Colorimetry	0.0022 U L^−1^ 0.011 U L^−1^	-	-	90	[24]
CTAB@AuNPs/ATP / Ca^2+^ / Pb^2+^	ALP	100–600 U L^−1^ 5–100 U L^−1^ 0.2–20 U L^−1^	Colorimetry	10 U L^−1^ 3.5 U L^−1^ 0.1 U L^−1^	-	-	55	[91]
DNAzyme-AuNP/HCR/GO	ALP	0.2 to 10 U L^−1^	Fluorescence	0.144 U L^−1^	-	-	-	[92]

From an analytical standpoint, analyte-mediated assays usually offer improved sensitivity as compared to those relying on pre-formed nanoparticles (Table 1). An explanation may be that the transition from the ionic/elemental/molecular state to the nanostructured state induces much more intense physicochemical changes, as compared to those obtained from the interaction between nanomaterials, thus offering improved sensitivity.

One of the most salient features of analyte-mediated nanoparticle assays is that they rely on the affinity of the target analytes for the precursor constituents of the nanomaterials. This feature offers two significant benefits; the first is that it minimizes non-specific interactions with non-target analytes affording improved selectivity, compared to assays that use pre-synthesized nanoparticles, which are more reactive against matrix components due to the high surface area and the chemical surface functionalization that is usually necessary to ensure their stability. Another benefit is the exploitation of reaction and sensing mechanisms which are not easily feasible with pre-formed nanomaterials. Multi-analyte assays, based on a single reaction pathway [25, 60, 67, 73, 79], the photochemically assisted synthesis of nanoparticles by dissolved organic matter [40], and the in situ formation of fluorescent nanomaterials as a result of complex biochemical reactions [25] are a few characteristic examples.

Although many advantages stem from the direct interaction of the analytes with the precursor materials, such interactions are not feasible or straightforward for all analytes. This restriction limits the applications of analyte-mediated nanoparticle assays as compared to sensing strategies that use pre-formed nanomaterials and can be tailored to the needs of the analysis (for example, by modifying nanoparticles with specific receptor molecules). Another disadvantage of analyte-mediated nanoparticle assays is that they usually involve several steps to accomplish a specific sequence of reactions that lead to signal emission. Each reaction needs a different time to be completed which does not facilitate the unattended operation of the assays. For the same reason, the analysis time is often longer than methods that use pre-synthesized nanoparticles (Table 1). Therefore, automation of analyte-mediated nanoparticle assays should be more complicated and require more advanced liquid handling operations.

## Summary and outlook

The analyte-mediated formation and enlargement of nanoparticles is a versatile platform for chemical and biochemical sensing. The sensing motif for analyte recognition is based on the particular chemistry and affinity of the precursor materials of the nanoparticles, while signal transduction is performed by exploiting the unique properties of the nanoparticles that are formed in situ during analysis. Although the pre-requisite for analyte interaction with one of the precursor materials brings some limitations (since not all analytes can interact with the precursor materials of the nanoparticles), a large plethora of assays have been developed for the determination of organic and inorganic compounds and macromolecules of biological, environmental, and food interest. Automation of experimental procedures and miniaturization to reduce reagent consumption in this approach are currently underdeveloped, but they could contribute to further avail its opportunities in portable analytical systems. Such systems are at the forefront of analytical research, and analyte-mediated nanoparticle assays offer a unique sensing medium with high sensitivity and exceptional selectivity. The immobilization of reagents onto surfaces and solid supports could assist both in automation and miniaturization and open new fields for research and development. Not least, the resolution of the misconception that this sensing strategy is similar to standard nanoparticle-based assays can instigate discoveries in chemical and biological sensing.

## Abbreviations

AChE: Acetylcholinesterase
ACTh: Acetylthiocholine
AdoHcy: S-adenosyl-L-homocysteine
AgNPs: Silver nanoparticles
AHCY: S-Adenosyl-L-homocysteine hydrolase
ALP: Alkaline phosphatase
APP: 4-Aminophenol phosphate
ATP: Adenosine triphosphate
Au NBPs: Gold nanobipyramids
AuNPs: Gold nanoparticles
AuNRs: Gold nanorods
Au NS: Gold nanostars
BSA: Bovine serum albumin
C-dots: Carbon dots
CeNPs: Ceria nanoparticles
CTA: Chromotropic acid
CUPRAC: Cupric reducing antioxidant capacity
Cys: Cysteine
DAMO: N-[3-(trimethoxysilyl)propyl]ethylenediamine
DCNP: Diethyl cyanophosphonate
DMAPE: 2-[2-(Dimethylamino)phenyl] ethanol
DNA: Deoxyribonucleic acid
DOM: Dissolved organic matter
ELISA: Enzyme-linked immunosorbent assay
FRAP: Ferric reducing antioxidant power
GCN-Cu NFs: Graphitic carbon nitride-Cu nanoflowers
GO: Graphene oxide
GOX: Glucose oxidase
GR: Glutathione reductase
GSH: Glutathione
GSSG: Glutathione disulfide
HCR: Hybridization chain reaction
HCy: Homocysteine
HPTS: 8-Hydroxypyrene-1,3,6-trisulfonic acid
IgG: Immunoglobulin G
LDH: Lactate dehydrogenase
L-DOPA: Levodopa
MGL: Methionine γ-lyase
NADH: Nicotinamide adenine dinucleotide
NADPH: Nicotinamide adenine dinucleotide phosphate
QDs: Quantum dots
OP: Organophosphorus
OTA: Ochratoxin A
PCR: Polymerase chain reaction
PON1: Serum paraoxonase
PSA: Prostate-specific antigen
PTA: S-phenyl thioacetate
Si CNPs: Silicon-containing nanoparticles
SiNPs: Silica nanoparticles
SPR: Surface plasmon resonance
TCh: Thiocholine
TYRase: Tyrosinase
VS-CDs: Vinyl sulfone-carbon dots
